# Uterine artery embolization for the treatment of symptomatic myomas in Brazilian women

**DOI:** 10.1590/S1516-31802003000500002

**Published:** 2003-09-01

**Authors:** Maurício Sena-Martins, Cecilia Maria Roteli-Martins, Valdir Tadini, Gustavo Antonio de Souza, Nestor Kisilevzky, Felipe Lazar

**Keywords:** Therapeutic embolization, Myoma, Uterine neoplasms, Uterine diseases, Treatment outcome, Embolização terapêutica, Mioma, Neoplasias dos genitais femininos, Útero, Resultado de tratamento

## Abstract

**CONTEXT:**

Uterine myomas are benign tumors that mostly occur in women of reproductive age at a frequency ranging from 20 to 25%. The symptoms are increased menstrual flow, pain and compressive signs. New treatments have been proposed and uterine artery embolization is one of them.

**OBJECTIVE:**

To evaluate the effects of treatment by embolization of the uterine artery, in women with symptomatic myomas. Uterine and dominant myoma volumes and the major symptoms were evaluated before treatment and 12 weeks later.

**TYPE OF STUDY:**

Open clinical trial.

**SETTING:**

A tertiary-care women's hospital.

**PARTICIPANTS:**

The study was conducted on 32 women with symptomatic single or multiple myomas of the uterine body, seen at the outpatient unit from May 2000 to September 2001.

**MAIN MEASUREMENTS:**

The patients were submitted to gynecological examination and abdominal and endovaginal pelvic ultrasonography, and the examinations were repeated 12 weeks after the first procedure. Uterine artery embolization using PVA (polyvinyl alcohol) particles of 355-700 μ was performed by catheterization of the right femoral artery in 30 women and by bilateral catheterization in two.

**RESULTS:**

Before embolization, the mean uterine volume of the 32 women was 455 cm^3^ and the mean volume of the dominant myoma was 150 cm^3^. Twelve weeks after embolization, the mean uterine volume was 256 cm and the mean volume of the dominant myoma was 91 cm^3^, with p < 0.01 in both cases. Twelve weeks after the treatment, all the women answered a questionnaire, which showed that 71% had improvement in menstrual regularity, 90% decreased menstrual volume and 81% shortened menstrual duration. The most frequent immediate post-procedure symptoms, established as complications, were pain (100%) and fatigue (34%). One woman had myoma degeneration and was submitted to myomectomy.

**CONCLUSION:**

The significant reduction in uterine and dominant myoma volume confirms the validity of the treatment of symptomatic myomas by the technique of uterine artery embolization in Brazilian women. There was significant reduction in menstrual flow and duration, as well as better cycle regularity in the women studied. The few adverse effects observed in the sample studied mainly involved pain immediately after embolization.

## RESUMO

**CONTEXTO:**

Miomas uterinos são tumores benignos que ocorrem na idade reprodutiva das mulheres com uma freqüência que varia entre 20% a 25%. Quando presentes os sintomas, estes se caracterizam por aumento do fluxo menstrual, dor e sinais de compressão. Têm sido propostas novas formas de tratamento, destacando-se a embolização das artérias uterinas.

**OBJETIVO:**

Avaliar os efeitos do tratamento com embolização das artérias uterinas em mulheres portadoras de miomas sintomáticos e os volumes uterino e do mioma dominante, antes e após 12 semanas após o procedimento.

**TIPO DE ESTUDO:**

Ensaio clínico aberto.

**LOCAL:**

Hospital Leonor Mendes de Barros, São Paulo, Brasil.

**PARTICIPANTES:**

32 mulheres com diagnóstico de mioma sintomático, único ou múltiplo, do corpo uterino, atendidas no Ambulatório de Ginecologia do Hospital Leonor Mendes de Barros, entre maio de 2000 e setembro de 2001.

**VARIÁVEIS ESTUDADAS:**

As mulheres foram submetidas a exame ginecológico seguido de ultra-sonografia abdominal, pélvica e endovaginal, repetidos 12 semanas após o procedimento. A embolização da artéria uterina com PVA (partículas de álcool polivinil de 355 a 700 μ) foi realizada através da cateterização da artéria femoral direita em 30 mulheres e, em duas mulheres, a cateterização foi bilateral.

**RESULTADOS:**

A média do volume uterino de 32 mulheres antes da embolização foi 455 cm^3^ e a média do volume do mioma dominante foi de 150 cm^3^ antes da embolização. Após 12 semanas de embolização, média do volume uterino e do mioma dominante foram 256 cm^3^ e 91 cm^3^ respectivamente. A redução dos volumes uterino e do mioma dominante foi estatisticamente significativa (p < 0,01) quando usado o teste de Wilcoxon. Doze semanas após o tratamento, questionário respondido por todas as mulheres mostrou melhor regularidade menstrual em 71%, redução do volume menstrual em 90% e menor duração em 81%. As complicações imediatas mais freqüentes foram dor e fadiga. Apenas em uma mulher ocorreu degeneração do mioma.

**CONCLUSÃO:**

A significativa diminuição dos volumes uterinos e dos miomas dominantes comprova a validade do tratamento dos miomas sintomáticos pela técnica de embolização das artérias uterinas. Houve uma redução significativa do fluxo e da duração menstrual, assim como uma melhor regularidade do ciclo nas mulheres estudadas. Nesta amostra, a dor imediata após a embolização foi o principal evento adverso observado.

## INTRODUCTION

Uterine myomas are benign tumors that mostly occur in women of reproductive age at a frequency ranging from 20 to 25% and are three times more common during the preclimacteric period.^1^ The dimensions of the tumors vary widely from 1 mm to 20 cm or more and their prevalence is difficult to estimate in most cases. Macro and microscopic examination of the uterus in specimens for anatomopathological study has shown a prevalence of 77%.^2^ Myomas occur especially among nulliparous obese black women with a family history of uterine myomatosis and hyperestrogenic syndromes, and they reach a considerable size in 50% of these women.^1^

Although most women with myomas are asymptomatic, complaints of abnormal uterine bleeding characterized by increased menstrual flow volume, pain and symptoms of adjacent organ compression are the most frequent symptoms, occurring at rates of 10 to 40%.^3^ The estimated annual cost of the treatment of uterine myomatosis is close to three billion dollars in the United States, excluding the expenses related to morbidity and missed days of work by the women. Furthermore, inter-currences related to myomas cause a higher rate of hospital stay than Aids, breast or prostate cancer, dementia, cirrhosis, and epilepsy.^4^

The conservative treatment used depends on factors such as number and size of the myomas, intensity of symptoms, and the woman's age and wish to reproduce. The purpose of this type of treatment is to minimize the severity of bleeding and reduce the volume of the tumors. Such procedures are also used before surgery for myomectomy and hysterectomy.^5^

In Paris in 1992, Ravina et al. were the first to use embolization of the uterine arteries specifically for the treatment of myomas, and the results of their study were published in 1995. Their approach was based on the observation of symptom improvement among women who were submitted to this procedure in order to reduce intraoperative bleeding before myomectomy.^6^

In the United States, Goodwin et al. (1997) were the first to report on this procedure applied to eleven women with an average age of 44 years who had been refractory to hormone therapy or myomectomy. These authors used PVA (polyvinyl alcohol) particles of 500-700 μ.^7^

Worthington-Kirsh et al. (1998) analyzed 53 women with menorrhagic and/or compressive symptoms, almost all of whom were successfully treated with arterial embolization.^8^ Recently, McLucas (2001) found similar results in 167 patients submitted to uterine artery embolization. In a literature review, Hurst et al. (2000) concluded that the use of uterine artery embolization is increasing as an alternative for the treatment of women with uterine fibroids who refuse surgery, despite its lack of long-term efficacy and maintenance of future fertility. They suggest further well-controlled trials to establish the safety and efficacy of this new treatment.^9,10^

Thus, there is evidence that uterine artery embolization may become a method of practical applicability and good acceptability among women who wish to preserve their uterus. Nonetheless, further studies are needed especially for evaluating the reproducibility of the technique and results obtained within the reality of women's healthcare in Brazil.

## METHODS

We selected women with a diagnosis of uterine myomatosis who wished to preserve their uteruses and who had been referred to the General Gynecology Outpatient Clinic of the Leonor Mendes de Barros Maternity Hospital. A total of 32 women with symptomatic myoma (mean age 40 years; range 27-51 years) were recruited for the study between August 2000 and May 2001. Since this is a tertiary care service, the women were referred by a doctor of the public health network (SUS), with or without a previous ultrasound examination. The patients were then submitted to a new gynecological and ultrasound examination for better determination of uterine volume and the volumes of the myomas. The average volumes of uterus and dominant fibroid were 455 cm^3^ and 150 cm^3^ prior to uterine artery embolization. Ten percent of the women were nulliparous, 25% primiparous and 65% had had two or more pregnancies. All women declared that they had menstrual disorders (volume or duration increase) and 12 weeks later all the women were asked about the volume, regularity and duration of bleeding in order to establish the magnitude of the menstrual disorder. Women with previous surgical treatment or recent clinical treatment and women with pedunculated myoma were excluded.

All the women were informed of the aim of the study and gave their consent. This study was approved by the local ethical committee from Hospital Leonor Mendes de Barros and also by the Brazilian National Research and Teaching Committee (CONEP), in compliance with the principles of the Helsinki Declaration, as reviewed in Hong Kong, and with adherence to good clinical practice. For statistical analysis, we used the Wilcoxon test.

### Techniques and examinations

#### Angiography and the method for uterine artery embolization

The procedures utilized Shimadzu digital angiography apparatus, model Digitex 2400. Prophylactic antibiotic treatment and analgesia were administered.

The femoral arteries were palpated in the inguinal region and a local anesthetic was applied by infiltrating 2% lidocaine in order to permit vessel puncture using the Seldinger technique.^11^ The right femoral artery was punctured for the introduction of a guide cannula. An introducer with a 5 F anti-reflux valve (1.66 m) was then inserted and the guide cannula was removed. A pigtail-type catheter was then inserted through the introducing guide to the level of the aortic bifurcation and 30 ml of radiological contrast (meglumine diatrizoate and sodium diatrizoate, Hypaque^→^) was injected for the angiographic study of the pelvis, in order to record the pelvic arterial map and identify the origin of the uterine arteries. The pigtail catheter was then replaced with a 5-F angiographic catheter of the femoral-visceral type (Dav, Cobra or Berestein).

The catheter was first advanced to the bifurcation of the aorta under fluoroscopic vision and positioned in the hypogastric artery. Selective catheterization was subsequently performed in the contralateral uterine artery. Contralateral uterine angiography was performed using approximately 10 ml of contrast in order to visualize the vessel architecture of the uterine artery and the distribution of its branches. The embolization material was prepared as follows: 1 PVA flask containing 355-500 μ or 500-700 μ particles was added to 10 ml contrast, 1 ml 0.9% physiological saline and one 40-mg ampoule of gentamicin. The material thus prepared was then injected through the angiographic catheter using small-volume syringes under fluoroscopic control, until vascular stasis and disappearance of the arched branches and the branches originating from the main trunk of the uterine artery were observed. A post-embolization angiographic check was performed at that time and documented on radiographic film. Using a procedure similar to that described above, ipsilateral catheterization of the hypogastric artery and uterine artery was performed via the Waltman maneuver, followed by ipsilateral uterine angiography, right artery embolization using PVA, and an angiographic check after embolization. The introducer and the catheter were removed and manual compression was performed over a mean time of 5 minutes until local homeostasis was obtained. Puncture of the left femoral artery was performed when the bilateral technique was absolutely necessary.

The women were instructed to return for follow-up and checks 1, 6 and 12 weeks after the procedure. Some women returned more frequently for signs or symptoms to be checked or for medication.

The patients admitted to the Leonor Mendes de Barros Maternity Hospital spent one to four days in the hospital (mean of two days) under the care of the investigating physician and doctors on shift duty, in order to obtain better control of pain or post-embolization syndrome. When the women returned for follow-up and checks 1, 6 and 12 weeks after the procedure, they gave responses to the continuation of the questionnaire, and were again submitted to clinical and ultrasound examination.

## RESULTS

**Figure 1:**
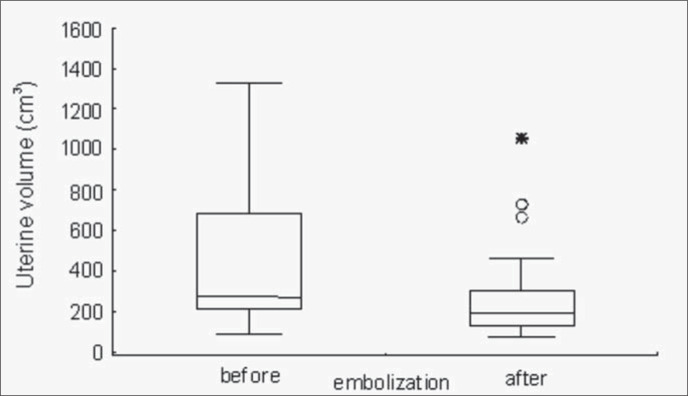
Comparison of the uterine volume before and after embolization. Wilcoxon test for paired samples, p < 0.01.

Uterine volume was significantly decreased after uterine artery embolization, in comparison with the volumes before uterine artery embolization (Figure 1). The smallest uterine volume was 84 cm^3^ before uterine artery embolization and 75 cm^3^ after uterine artery embolization. The largest uterine volume was 1,325 cm^3^ before uterine artery embolization and 1,055 cm^3^ after uterine artery embolization.

**Figure 2:**
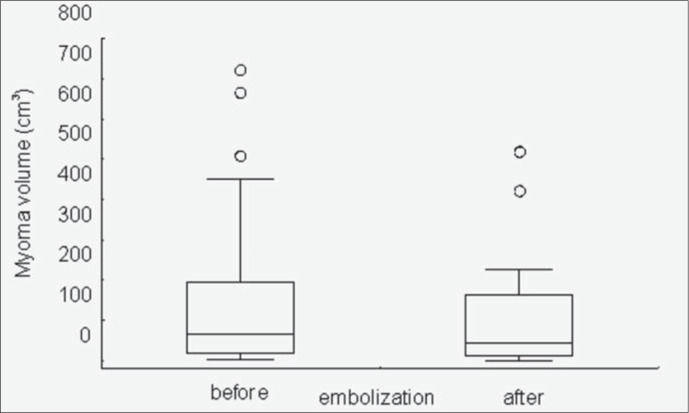
Comparison of the volumes of the dominant myoma before and after embolization. Wilcoxon test for paired samples, p < 0.01.

The difference in both the 25^th^ and 75^th^ quartiles of the dominant myoma volumes before and after uterine artery embolization was significant, as shown by the Wilcoxon test for paired samples (p < 0.01) (Figure 2). The volume of the dominant myomas was significantly decreased 12 weeks after uterine artery embolization. The smallest myoma size was 4 cm^3^ before uterine artery embolization, with total regression after the procedure. The largest myoma size was 722 cm^3^ before uterine artery embolization and 519 cm^3^ after uterine artery embolization. The values represented by circles in the graph were beyond the maximum value but still within the scale (Figure 2).

Of the 32 women submitted to uterine artery embolization and evaluated 12 weeks later, one was excluded because of myoma degeneration, and myomectomy was performed; 27 presented a mean of 42% reduction in uterine volume; while only 4 women presented an increase in mean uterine volume (6%) after uterine artery embolization. With respect to the dominant myoma, there was a reduction in volume in 27 women and an increase in 3. In one woman there was no change in the volume of the dominant myoma (Table 1).

**Table 1:** Percent variation in uterine and dominant myoma volumes 12 weeks after embolization*

		*Reduction in volume*				*Increase in volume*		
	n	mean	minimum	maximum	n	mean	minimum	maximum
		(%)	(%)	(%)		(%)	(%)	(%)
Uterus	27	41.6	9.4	73.4	4	5.6	2.4	12.1
Myoma	27	40.5	4.5	100	3	52.2	2.2	119.9

* *One woman was not included in this analysis and another one had no variation in myoma volume.*

In 22 women there was better menstrual regularity 12 weeks after the procedure, 28 presented a reduction in menstrual volume and 25 declared a shorter duration (Table 2). The most frequent immediate complications were pain and fatigue. Myoma degeneration occurred in one woman who had a pedunculated myoma immured in the small pelvis. The patient was hospitalized and properly treated via myomectomy, with a satisfactory course that had no complications or sequelae. Myoma elimination by the vaginal route occurred in two women. There were no infectious complications or allergies relating to contrast, antibiotics or anesthetic agents (Table 3).

**Table 2:** Frequency distribution of women submitted to artery embolization according to changes in menstrual rhythm, after a 12-week period

		Change in menstrual regularity	
	**n**		**%**
Equal	7		22.6
Regular	22		71.0
Irregular	2		6.4
		**Change in menstrual volume**	
	**n**		**%**
Equal	3		9.7
Lower	28		90.3
		**Change in menstrual duration**	
	**n**		**%**
Equal	6		19.4
Shorter	25		80.6
**Total**	**31**		**100**

*One woman (case 3) was not included in this analysis.*

**Table 3:** Complications occurring immediately and up to 12 weeks after uterine artery embolization in 32 women

	n	%
Post-embolization syndrome		
*Pain*	32/32	(100)
*Fever (> 37.5 °C)*	0/32	(0)
*Fatigue*	11/32	(34)
Degeneration	1/32	(3)
Infection	0/32	(0)
Myoma elimination	2/32	(6)
Puncturing accident (hematoma)	3/32	(9)
Allergy	0/32	(0)

## DISCUSSION

The main objective of the present investigation was to evaluate the characteristics of the uterus after uterine artery embolization in 32 Brazilian women with symptomatic myomas treated from August 2000 to September 2001.

The type of myomatosis may influence the decision to use the uterine artery embolization procedure in some selected cases. Multiple myomas for which the surgical approach is difficult, giant myomas with marked compressive symptoms, or clinically intractable myomas for which the only option would be hysterectomy even among young women, could be submitted to uterine artery embolization if the patient wished to keep her uterus.^12^ It should also be pointed out that abdominal or laparoscopic myomectomy, which could be another option for women with multiple myomas, presents complications such as adhesions, development of new myomas, and the possibility of conversion to hysterectomy.^13^ One of the patients in our series, a 27-year-old woman, presented a satisfactory result both in anatomical and symptomatic terms, a fact that has stimulated us to consider this alternative more frequently.

Most of the women submitted to uterine artery embolization presented a mean reduction in uterine volume and dominant myoma volume of about half the initial volume after 12 weeks. With respect to the major symptoms, the best result was the reduction in menstrual flow observed in almost all women in this series. Greater regularity of the menstrual cycle and an effective reduction in the number of days of menstruation were also observed.

In our study, the main symptom reported by the patients was an increase in menstrual flow, present in all cases, associated with pelvic pain in some cases (13%) or with increased abdominal volume (3%). These findings agree with those reported by most investigators, who stated that 85 to 100% of embolized women had menorrhagia as the major symptom.^6,8^

Our results are closely similar to those obtained at other centers, since we obtained a reduction in menstrual flow in 90% of our patients (28 with an effective decrease in flow and three with no changes), indicating good reproducibility of the current uterine artery embolization technique. In addition to menstrual flow, we believe that it is important to consider two more features concerning menstruation, i.e. regularity of the cycle and menstrual duration. Better regularity in these parameters was observed in 71% of cases, with seven women maintaining regular cycles and two continuing to have irregular cycles. There was also a decrease in the number of days of menstruation in 80% of the women, while no change in duration was observed in the remaining women.^14^

Twelve weeks after uterine artery embolization, two of the 32 women studied (one of them was excluded) presented amenorrhea. One was a 47-year-old severely hypertensive woman with no vasomotor symptoms and a follicle-stimulating hormone (FSH) level of 25.9 mIU/ml, and the other was a 50-year-old woman with FSH of 16.9 mIU/ml, who started to have regular cycles again. Ovarian failure after uterine artery embolization was first reported by Stringer et al. (2000). Spies et al. (2001) studied 63 patients after uterine artery embolization and concluded that, while most patients had no change in ovarian function as measured by basal FSH, for patients aged 45 or older there is an approximately 15% chance of an increase in basal FSH into the perimenopausal range. Hysterectomy was not necessary in any of our cases, confirming the studies cited earlier, but one woman was submitted to myomectomy because of undiagnosed pedunculated myoma.^15,16^

Another important aspect of our series was the low rate of complications during the examinations, such as vasospasms, and immediately after them, such as puncture hematomas. This reflected the excellence of the techniques used by the operating radiologist and the nursing team providing the postoperative care.

In the present study, uterine volume showed a mean decrease of 40% 12 weeks after uterine artery embolization, in agreement with most other studies. From the initial report by Ravina et al. (1995) up to current studies, the decreases in uterine volume found have ranged from 27 to 51%, most frequently corresponding to 40 to 44%.^6-8,17-23^

Bradley et al. (1998) reported the highest rate of reduction (51%), in eight women with symptomatic myomas that had a mean volume of 1,300 cm^3^ before embolization. This result can be better understood by considering two factors: volume-dependence, i.e. the larger the initial volume, the greater the percentage reduction after uterine artery embolization; and the use of a more sensitive method for the evaluation, i.e. nuclear magnetic resonance.^12^

Most of the women studied here had uterine volumes of less than 300 cm^3^ and we opted for the use of combined ultrasound when necessary, with evaluation performed by the same examiner and the same equipment before and after embolization, in order to reduce the inter-observer and equipment-dependent error.

In our sample, four women showed a mean increase of 5%. This slight, non-significant increase may be explained by the range of variation in reliability of the method and it was observed in uteri with volumes of less than 200 cm^3^. These results lead us to infer that the small differences in measurements observed 12 weeks after uterine artery embolization may have masked the evaluation of uteri of smaller volume in terms of percent variation in comparison with uteri of larger volume.

In our series, the four women who did not show a reduction in uterine volume, as mentioned earlier, also showed a significant improvement in the main symptom. These results were a little less expressive than those reported in most of the published studies, which showed a mean reduction of 43 to 60%.^7,17-19,21,23-27^ This may be explained by two factors, i.e. the characteristics of the uterus embolized in this study, which in most cases had a volume of less than 300 cm^3^; and the time when the evaluation was performed, which in our study was 12 weeks.

Of the 31 women studied here whose follow-up was concluded after 12 weeks (one was submitted to myomectomy before this time), four did not present a reduction in the dominant myoma. One of them continued to have a stationary volume, two showed a non-significant decrease, and the last showed a considerable increase from 236 to 519 cm^3^. This increase was possibly due to asymptomatic degeneration of the myoma, with no need for any intervention. There was an 18% reduction in uterine volume and, as a precaution-ary measure, the return visits of these patients were scheduled at higher frequency.

It is interesting to note that most of these women were already starting to present marked reduction in flow during the first menstrual episode. These data agree with those reported by Pelage et al. (2000) who observed improved menstrual flow in the first cycle in 92% of cases.^20^

In our sample, one woman was submitted to myomectomy eight weeks after uterine artery embolization because she presented hyaline degeneration in a voluminous and pedunculated myoma whose pedicle was not identified by clinical examination or ultra-sound because it was fixed to the smaller pelvis. Another woman eliminated myoma fragments by the vaginal route and was submitted to curettage of the uterus. In the same way as reported by Berkowitz et al. (1999) and Abbara et al. (1999), this patient also experienced strong cramps associated with slight bleeding, especially at the time of elimination. In our sample, all patients experienced pelvic pain of differing degrees after embolization, using the established pain protocol (treated with ketoprofen 100 mg IV, meperidine 100 mg). None of them used epidural anesthesia.^28,29^

Andersen et al. (2001) used, on average, three PVA flasks of particles measuring 355-500 μ in 62 women.^30^ We used, on average, two particle flasks, perhaps because of the elasticity of the use of different particle sizes, or because we established technical success when we observed the absence of flow and retention of contrast in the uterus, known as the parenchymatous phase of embolization, an event that sometimes varies from one report to another.^30^

From a technical viewpoint, we observed the importance of the gynecologist's participation in the judicious selection of cases, explaining the technique to be adopted for the patient, answering patients’ queries and, especially, transmitting security through his presence on the day of the procedure. In addition, the increasing experience and consequent greater skill of the operating radiologist may lead to more rapid embolization procedures, with gradually reduced exposure to radiation.

## CONCLUSION

In conclusion, the significant reduction in the uterine and dominant myoma volumes has confirmed the validity of the treatment of symptomatic myomas by uterine artery embolization. Among the symptoms that had been present before the procedure, there was significant reduction in menstrual flow and duration, as well as better regularity of the cycle in the women studied. Few adverse effects were observed, mainly represented by pain immediately after embolization, further indicating the feasibility of using this procedure for the treatment of selected cases of women with symptomatic myomas.
